# Iatrogenic Left Ventricle Outflow Tract Ventricular Tachycardia Following Transcatheter Aortic Valve Replacement: A Case Series

**DOI:** 10.14797/mdcvj.1284

**Published:** 2023-10-20

**Authors:** Ahmed Hassaan Qavi, Aditi Naniwadekar, Neeraj N. Shah

**Affiliations:** 1 East Carolina University Health Medical Center, Greenville, North Carolina, US

**Keywords:** left ventricular outflow tract, ventricular tachycardia, transcatheter aortic valve replacement

## Abstract

Focal left ventricular outflow tract ventricular tachycardia (LVOT-VT) is rarely reported following transcatheter aortic valve replacement (TAVR). Similarly, unexplained sudden cardiac death after TAVR also is rarely described and may be attributed to VT. We present two cases of patients who underwent TAVR and later presented with VT of suggested LVOT origin. Both patients were treated with amiodarone for suppression of VT.

## Introduction

Our first case presents a 70-year-old male with bicuspid aortic valve (Sievers type 0) who was found to have severe aortic valve stenosis. He underwent uncomplicated transfemoral transcatheter aortic valve replacement (TAVR) with a 26-mm Edwards SAPIEN-3 Ultra valve (Edwards Lifesciences Corp). Five months later, he presented to the emergency room with dizziness and dyspnea. An electrocardiogram (ECG) suggested ventricular tachycardia (VT) originating from the left ventricular outflow tract (LVOT). Cardiac magnetic resonance imaging (MRI) revealed no fibrosis. The patient was treated with amiodarone with suppression of VT.

Our second case presents an 80-year-old male who was diagnosed with symptomatic severe aortic stenosis. He also underwent transfemoral TAVR with 26-mm Edwards SAPIEN-3 Ultra valve. He had complete heart block postoperatively, necessitating a pacemaker. Twelve hours later, he suffered sustained VT that appeared on ECG to originate from the LVOT. Coronary angiography showed patent coronary arteries. He was managed with amiodarone for suppression of VT, similar to the first patient.

## Background

Transcatheter aortic valve replacement is a therapy of choice for definitive treatment of older patients with severe symptomatic aortic stenosis.^1^ Conduction abnormalities and the need for pacemaker implantation following TAVR implantation are widely recognized complications.^2,3^ However, postprocedural ventricular arrythmias (VA) have rarely been reported.^2,4,5,6^ There remains a significant paucity of data on the incidence and prognostic significance of these tachyarrhythmias.^1^ We present two cases of focal outflow tract ventricular tachycardia after transcatheter aortic valve implantation—one occurring early and the other late.

## Case 1

A 70-year-old-male with hypertension, bicuspid aortic valve (Sievers type 0) and stable ascending aorta dilatation (4.1 cm) presented with progressive dyspnea due to severe aortic valve stenosis. Transthoracic echocardiography (TTE) revealed preserved left ventricular ejection fraction (LVEF), estimated aortic valve area (AVA) 0.8 cm^2^, peak aortic velocity 4.9 m/s, and a mean aortic gradient of 63 mm Hg. The patient was evaluated and deemed appropriate for TAVR. Preimplantation balloon-aortic valvuloplasty (pre-BAV) was performed with a 20-mm Z-Med-II balloon (Braun Interventional Systems Inc.) to facilitate delivery of the transcatheter valve system followed by successful transfemoral TAVR with 26-mm Edwards SAPIEN-3 Ultra valve (Video 1). This resulted in marked improvement in symptoms and functional capacity at 1 month follow-up.

**Video 1:** In Case 1, pre-BAV was performed with a 20-mm Z-Med-II balloon to facilitate delivery of the transcatheter valve system followed by successful transfemoral transcatheter aortic valve replacement with 26-mm Edwards SAPIEN-3 Ultra valve; see also at https://youtu.be/tQL--I3zPY8. pre-BAV: pre-implantation balloon-aortic valvuloplasty

**Figure 1:**
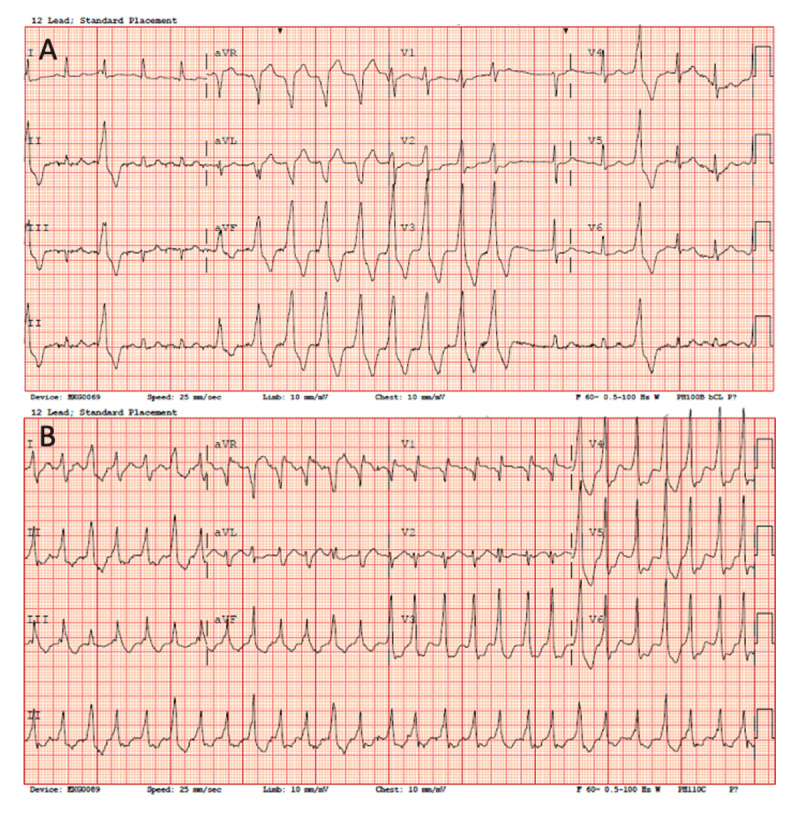
**(A)** In case 1, 12-lead electrocardiogram shows inferior axis, QS pattern in leads aVR and aVL, and precordial transition earlier than V3, suggestive of left ventricular outflow tract origin. **(B)** Case 2 shows QR pattern in V1, inferior axis, voltage ratio of lead II/III > 1, and precordial transition in V3, suggestive of left ventricular outflow tract origin.

Five months later, the patient presented to the emergency room with acute onset of dyspnea and dizziness. Cardiac telemetry showed multiple runs of nonsustained ventricular tachycardia (NSVT) at 210 to 220 beats per minute (bpm). Electrocardiographic morphology of the NSVT showed inferior axis, QS pattern in leads aVR and aVL, and precordial transition earlier than V3, suggestive of LVOT origin (Figure 1A). Repeat TTE showed a normal LVEF and well-functioning TAVR valve with no paravalvular leak. Cardiac MRI demonstrated no fibrosis or scar.

Treatment with intravenous amiodarone successfully suppressed the VT. The patient was transitioned to oral amiodarone and suffered no further arrhythmias. Cardiac event monitor showed no arrhythmias at 1 month follow-up. Oral amiodarone was discontinued, followed by implantable loop recorder insertion for prolonged monitoring. At 16-month follow-up, implantable loop recorder interrogation revealed no further VT events.

## Case 2

An 80-year-old male with hypertension presented with progressive dyspnea and fatigue. TTE revealed severe aortic valve stenosis (estimated AVA 0.7 cm^2^, peak aortic velocity 4.5 m/s, mean aortic gradient 38 mm Hg) and LVEF 45%. ECG showed sinus rhythm at 61 bpm and prolonged PR interval. Given the patient’s age and comorbidities, he was evaluated and found appropriate for TAVR. He successfully underwent pre-BAV with a 20-mm Z-Med-II balloon followed by transfemoral TAVR with a 26-mm Edwards SAPIEN-3 Ultra valve (Video 2). Postoperative course was complicated by asymptomatic sinus bradycardia with episodes of second-degree Mobitz type-I block. He was discharged on the third postoperative day with a cardiac event monitor.

**Video 2:** In Case 2, pre-BAV with a 20-mm Z-Med-II balloon followed by transfemoral transcatheter aortic valve replacement with a 26-mm Edwards SAPIEN-3 Ultra valve; see also at https://youtu.be/PtCRCwWqjJQ. pre-BAV: pre-implantation balloon-aortic valvuloplasty

The patient returned to the emergency room the following day after the heart monitor company alerted him of a new arrhythmia. This was confirmed as a complete heart block with junctional escape rhythm that culminated in implantation of a conventional dual-chamber permanent pacemaker. Twelve hours later, the patient had an hour-long episode of sustained VT most likely in the LVOT region as manifested by a QR pattern in V1, inferior axis, voltage ratio of lead II/III > 1, and precordial transition in V3 (Figure 1B). Repeat TTE showed a well-functioning TAVR valve, no paravalvular leak, and interval LVEF decline to 35-40%. Invasive coronary angiography demonstrated patent coronary arteries. He was treated with intravenous amiodarone and had recurrent short NSVT episodes with eventual suppression. He was discharged on long-term oral amiodarone and beta-blocker. At 6-month follow-up, there were no further VT events on pacemaker interrogation. His oral amiodarone was continued.

## Discussion

Ventricular arrhythmias following TAVR are rarely reported despite increasing pervasiveness of the procedure.^1,2,3^ To date, only three cases of LVOT-VT after TAVR have been reported.^2,4,5,6^ We describe two such cases in patients undergoing TAVR with a balloon-expandable valve. One patient presented several months later and the other presented within the first week of implantation. ECG features of arrhythmias in both patients were congruent with LVOT-VT. The first patient had NSVT; however, the second patient had sustained VT. These events were not associated with ischemia, electrolyte abnormalities, or drugs.

Outflow tract VT is usually related to cyclic adenosine monophosphate mediated triggered activity; however, non-reentrant mechanisms have rarely been reported.^2,3^ Mechanisms to explain post TAVR LVOT-VT include disease process affecting the valve and adjacent area, direct mechanical pressure of balloon or valve stent against LVOT, change in LVOT geometry, periaortic scar, or a yet unknown mechanism from an unrelated substrate.^2,3,4^ Our first patient had VT several months later, making acute tissue injury less likely, whereas the onset of VT in our second patient was more acute. One can argue that in the first patient there may have been subclinical LVOT injury post-TAVR, which healed and resulted in a small focal scar that served as substrate for VT; however, cardiac MRI did not reveal any obvious scar. Given successful suppression of VT with antiarrhythmic therapy (in both cases), we did not perform electrophysiologic mapping in order to avoid going through the TAVR prosthesis. Furthermore, both patients remained arrhythmia-free during extended follow-up. There have, however, been prior reported cases in which ablation was successfully performed to terminate the VA and may be considered as a viable alternate.^2,4^

New-onset ventricular arrhythmias after TAVR are rare, with NSVT found in only 6% of patients and only one prior report of sustained VT.^4^ Another study showed that the incidence of VT decreased from 9.6% prior to TAVR to 4.8% at 1 month and 2% at 1-year post-TAVR.^7^ Whether VAs are truly new onset or a result of unmasking of prior pathology in the left ventricle is unknown, as is the long-term prognosis of these events.^1,3^

In our series of two patients, the LVOT-VT events occurred after a few days to several months following implantation of balloon-expandable valves, were transient and self-limited, were successfully treated, were suppressed with conservative management with antiarrhythmic therapy, and did not recur during extended follow-up. This supports the hypothesis of transient/subclinical LVOT injury post-TAVR, resulting in either irritation or scarring in the region of distal His-Purkinje/proximal left bundle area as the underlying mechanism behind both events. Even so, the presence of unexplained sudden death post-TAVR and available data of mortality associated with ventricular arrhythmias emphasize the need for careful monitoring and focused studies evaluating the risk factors and clinical impact of ventricular arrhythmias post-TAVR.^1,2^

## Conclusion

This series presented two cases of LVOT-VT following TAVR, both successfully treated with antiarrhythmic therapy without recurrence during extended follow-up. However, it is important to note that unexplained sudden cardiac death after TAVR may be attributable to VT, necessitating awareness of this rare association.
